# "The Hospital" Nursing Section

**Published:** 1906-06-16

**Authors:** 


### The Hospital.

### Nursing Section.

Contributions for this Section of "The Hospital" should be addressed to the Editor, "The Hospital" Nursing Section, 28 & 29 Southampton Street, Strand, London, W.C.

No. 1,029.—Vol. XL. SATURDAY, JUNE 16, 1906.

### Notes on News from the Nursing World.

### ROYALTY AND THE NURSING OF EUROPEANS IN INDIA.

It is only a fortnight ago that we mentioned the needs of the Up-Country Nursing Association for Europeans in India. We learn with pleasure that the Prince and Princess of Wales have each given £25 towards its funds, and that other special donations include contributions from the past and present Secretary of State for Foreign Affairs. With respect to the donations of the Prince and Princess, we have no doubt that during their recent visit to India the importance of increasing the supply of trained nurses in the Dependency was brought home to them. In any case we trust that their timely help will afford a much-desired impetus to a movement which appeals in particular to all who have links with India.

### THE GOVERNMENT AND STATE REGISTRATION.

The Lord President of the Council is receiving at noon to-day, Thursday, an important deputation against the Registration of Nurses. The deputation consists of four members representing the Central Hospitals Council, namely, Mr. H. A. Harben, the Hon. Sydney Holland, Dr. Allchin, and Dr. Kingston Fowler; and there will also be present Sir Thomas Barlow, Sir Frederick Treves, the Duchess of Bedford, the Marchioness of Salisbury, the Countess of Pembroke, Viscountess St. Aldwyn, Lord Rayleigh, Lady Northcliffe, the Hon. Maude Stanley, Mrs. J. A. Spender, the matrons of St. Thomas's, the London, King's College, St. Mary's, and the Seamen's Hospitals, St. Marylebone Infirmary, the Royal Infirmary, Bristol, the Royal Derbyshire Infirmary, Radcliffe Infirmary, Oxford, and Addenbrooke's Hospital, Cambridge. Dr. Lauriston Shaw states in the *Times* of June 13th that the deputation from the Central Hospital Council to be received by Lord Crewe is not deputed to oppose the registration of nurses, but to suggest the establishment of an official directory.

### THE QUEEN'S NURSE AND THE SICK CHILD—OFFICIAL CONTRADICTION.

According to the statements which appeared in the daily press last Thursday, the Queen's Nurse at Holywood, Belfast, refused milk to a sick child because it was illegitimate. As soon as the general superintendent of Queen Victoria's Jubilee Institute for Nurses saw the statements she asked for a full report of the case from the Irish superintendent. The Irish superintendent replied that she herself only became acquainted with the difficulty by means of the press. She enclosed a report of the Holywood Association, and the rules are identical with those of the Institute. No. 12, the only one which refers to any question of relief, is to the effect that the nurse cannot give either nourishment, clothes, or medicine to patients unless in case of urgent necessity. As to the main point, there is absolutely no rule in the Queen's Institute or at Holywood by which nourishment or treatment would be withheld from a sick person or any question of creed, birth, or any other cause. We understand that Miss Lamont, the Irish superintendent, has gone to Belfast to inquire into the circumstances of the case still more fully. The matter also came before the Nursing Committee of the Institute on Wednesday afternoon, with the result that the following official contradiction has been sent to us by the Secretary:—"The attention of the Committee of Queen Victoria's Jubilee Institute for Nurses has been drawn to a statement in the press that nourishment was refused by a Queen's Nurse to an illegitimate child on account of the stigma attached to its birth. The Committee wish to contradict the impression erroneously conveyed that there is any regulation in the rules of the Queen's Institute which could lead to such an action on the part of a Queen's Nurse. Queen's Nurses are allowed to give relief and nourishment only in time of emergency, and are required to report any such cases at once to the proper authority, systematic relief-giving not being part of their duty. The services of the Queen's Nurses and other benefits available through them are given to patients freely according to the needs of sickness apart from the consideration of any other circumstances."

### "MENTAL SCIENCE" NURSES.

Attention is called by a Massachusetts paper to some recent disclosures regarding a "mental science" trained nurse who recently substituted her own treatment based on a fantastic system of spiritual therapeutics for the prescription of the physician. Our contemporary, which seems to think that a considerable number of trained nurses in America have been infected with "similar pseudo-scientific nonsense," goes on to say that, "if under cover of the special badge of fitness worn by the licensed members of the nursing profession, the sick are apt to be placed in charge of some fad-made creature who throws the prescribed medicine out of the window, and substitutes for it prayer and other mental exercises, a return to the old method of home nursing is directly in order." In other words, it is evidently feared in the United States that registration may tend to put terrible power into the hands of women who are roughly, but not inaccurately, described as "dangerous cranks." We lately observed that there is no room in the nursing world on this side of the Atlantic for women who believe in the practice of faith-healing as a substitute for medical treatment; and we are glad that on the other side the urgent necessity for weeding "mental science" nurses out of the ranks is recognised.

### A CHILDREN'S NURSES' COLUMN.

In consequence of the increasing demand for children's nurses who are fully qualified, and the difficulty experienced in obtaining them, a special column has been opened by the manager of The Hospital for the purpose of inserting intimations from ladies who desire to secure the services of responsible nurses, and from those who have prepared themselves for the work. It is well known that there is a certain proportion of nurses who, from one cause or another, have been unable to complete their three years' training, and yet do not lack the knowledge and the sense of responsibility which cannot be valued too highly by a young inexperienced mother, or by one who has frequently to leave her children to the charge of others. The ordinary domestic nurse seldom possesses these qualifications, and we hope that by bringing the mother and the trained nurse together in respect to the care of children, we may be of benefit to both. Full particulars as to the conditions on which the announcements are made in the special column will be found elsewhere.

### GUARDIANS AND SALARIES.

At a meeting of the Fylde Guardians last week a recommendation that a trained nurse be appointed to the Workhouse Hospital at a commencing salary of £25 a year, was met by an amendment that the amount should be £30. Miss Johnson, who moved the amendment, stated that seventeen years ago, when she came out of her training school, she received £30 a year on her first appointment; and she expressed the opinion that in view of the different character of the nurse nowadays £25 was far too little. When one of the Guardians defended the recommendation on the ground that the nurses at Whittingham Asylum receive £25 a year, she rejoined, with unanswerable force, that the asylum workers at Whittingham are not trained nurses. Moreover, another Guardian not only agreed with Miss Johnson, but declared that "if he had a daughter who applied for a situation at £25, after spending a number of years in training, he would knock her neck out before he would let her take the place." Neither of these protests, however, availed, and the recommendation was carried. The Fylde Guardians have not, therefore, even come into line with those of Harrogate, who recently increased their scale of pay from £23 with annual advances of £1, to £26 with annual advances of £2. The wisdom of this course was shown in a conclusive manner. An advertisement for a nurse on the old scale brought three replies and no suitable candidate; an advertisement for one on the revised scale produced 24 replies, and many suitable candidates.

### THE ADVANTAGE OF WRITTEN AGREEMENTS.

A judgment of some interest has been given by the Judge of the Liverpool Court of Passage. The plaintiff was Miss Palmer, a nurse, who sued a former patient for fourteen guineas, her fees for treatment in her nursing home. The plaintiff alleged that the charge of £6 6s. per week was in accordance with the arrangement made; but the defendant had only paid according to the rate of four guineas per week, as she affirmed that someone else had informed her that would be the rate. The defendant did not appear and it was stated that she had to leave England on the previous night in order to join her husband in China. In her absence the Judge allowed the claim of the plaintiff for the full amount. The judgment seems fair enough in the circumstances, but it would save disputes if in such cases the terms were reduced to writing.

### SICK LEAVE AND SALARY.

The Irish Poor-law Association have been discussing the case of a nurse attached to the Callan Union Infirmary, who, at the end of her holidays, was ordered by her medical man an additional five weeks' sick leave. The Guardians in paying her salary deducted £9 2s., the amount paid to her substitute, and the Association adopted a resolution agreeing to indemnify her for any law costs she may incur in taking legal proceedings to recover her salary. It was stated that previous to her illness the nurse had been overworked owing to the large number of typhoid cases in the Infirmary. Speaking generally, we think that if the illness of a nurse is directly due to over-work in the discharge of her duties, Guardians ought to treat her liberally, and if she has been in their service for a considerable period, and the prolonged holiday has restored her to full working powers, it is both just and politic to waive the question. But it can be understood that if the health of a nurse breaks down soon after she enters upon her duties, or she seems constitutionally delicate, the Guardians, as trustees of the ratepayers' money, are unwilling to pay two nurses at the same time for the same work.

### AN EAST-END FUNCTION.

The hard work of all those who organised the successful Fête and Sale of Work at the Home of the Shoreditch and Bethnal Green District Nursing Association on Tuesday was rewarded by brilliant sunshine, a large gathering, the presence of Royalty, and, we hope, a substantial sum to add to the funds. The small garden at 80 Nichol Square was crowded with visitors awaiting the arrival of Princess Victoria of Schleswig-Holstein, the interval being beguilded by the Highbury Ladies' String Band. Princess Victoria arrived about 3.30, looking very cool and pleasant in a dark blue muslin over white, with a large white feather boa. Miss Winnie Oliver having presented a bouquet of pink roses, the Rev. H. S. Eck gave a short address of welcome. Thirty-two purses were then presented by small people of varying ages from two years old and upwards, the Princess proceeded to visit the stalls and made purchases at all of them, ending up with an iced cake from the nurses' stall for her tea. Before leaving she went over the home and expressed her satisfaction with all its arrangements. The downstairs rooms were, of course, transformed for the time being into stalls and tea-rooms. The nurses' stall, with its home-made cakes, jams, and other provisions, was a centre of attraction. This was the first function of the kind they had attempted, and Miss Böge, the lady superintendent, has no cause to be dissatisfied with the result even if the amount cleared is not as large as she desired.

### THE PENSION FUND AND NEWCASTLE NURSES.

An address on the aims and objects of the Royal National Pension Fund for Nurses will be delivered at the Royal Infirmary, Newcastle-on-Tyne, by the Secretary, Mr. Louis H. M. Dick, on Wednesday, June 20, at 6.30 p.m. The library of the institution has courteously been lent for the purpose by the authorities, and all nurses in the district are cordially invited to attend. By the kindness of the matron, Miss Aston, light refreshments will be served at the close of the meeting. Mention of Newcastle recalls the fact that the trustees of the Heath Bequest pay one-fourth of the premiums contributed to the Pension Fund by nurses attached to certain institutions in that city, providing that such proportion does not exceed £2 per annum on behalf of each nurse.

### SOCIAL AMENITIES AT THE ANTIPODES.

The business conference of the Council of the Royal Victorian Trained Nurses' Association, on Friday, April 27, was preceded by a pleasant social function in the shape of a dinner to the delegates appointed to represent the Australasian Trained Nurses' Association at the Conference. About 20 guests were present, and some excellent speeches were delivered, a hope being expressed by the visitors that they might have the gratification of entertaining the members of the Victorian Association next year.

### CALIFORNIAN NURSES IN NEED.

An appeal is being made by the Californian Nurses' State Association for help for the graduate nurses on duty at the various camps, who at the time of the San Francisco earthquake lost everything they owned except the uniform and underclothing in which they left the burning and falling buildings with their patients. In spite of the large amounts sent to the relief fund, the nurses, it is affirmed, have as yet received no benefit from it except food. An "endless-chain collection" has, accordingly, been started on their behalf.

### PECKHAM NURSING ASSOCIATION.

Lady Duckworth opened a Sale of Work in aid of the funds of this Association on Tuesday last. Sir Dyce Duckworth had intended to be present but he was prevented from attending. The nurses of the Association, which was founded six years ago, paid 13,137 visits to 690 patients last year. The management is entirely honorary, only the nurses receiving payment for services. The rapid increase of the work has created a deficit which the churches of the district of all denominations are uniting in an effort to cover.

### A SECOND SISTER FOR OBERAMMERGAU.

We are pleased to learn that Miss Edith Milner was able last week to present to the Burgomaster of Oberammergau a sum of £500 to provide for the maintenance of a second resident nursing sister in the Cottage Hospital of the village. Thanks largely to the efforts of Miss Milner, whom we gladly assisted to obtain the required publicity, this sum of money has been subscribed by English people as a testimony to the deep impression made upon them by witnessing the Oberammergau Passion Play. The list of subscribers was headed by King Edward, who, as Prince of Wales, saw the play in 1871; and the Duke of Connaught, who has seen it on two occasions, also contributed. It is extremely satisfactory to know that the needs of the sick poor in the neighbourhood have thus been met.

### NURSES' UNION.

On Tuesday afternoon last week the members of the Nurses' Union were entertained by Mrs. Grier in her garden at Grove House, Regent's Park. The proceedings commenced with a Bible reading by Miss Dashwood, Hon. Secretary of the Union, to which the nurses, in comfortable easy chairs, listened with much interest. Subsequently, they walked about the delightful garden, and when they were tired, returned to the drawing-room, where tea was served. The "At Home" lasted from 2.30 to 4, but it came to an end all too soon for the guests, who thoroughly enjoyed the rest and change so kindly provided for them.

### A PROSPEROUS PROVINCIAL ASSOCIATION.

The annual meeting of the Royal Derby and Derbyshire Nursing and Sanitary Association was held last Friday. A very satisfactory report was submitted, the financial statement showing an ample credit balance. The nurses have again received, in addition to the usual bonus of 10 per cent. upon their earnings, a special bonus of £365, and the balance of £332 has been added to the contingent fund for old and disabled nurses, which now amounts to £2,881. To the Nightingale Home purchase and furnishing Fund has been added £300 as training fees for midwifery nurses. As to the work of the nurses, of whom there are 116, 70 are engaged in private nursing, there are 18 district nurses, and 6 pupils, 5 college nurses, 1 staff nurse, 1 night nurse, and 2 pupils engaged at the "Home of Rest," as well as 20 probationers who are being trained at various hospitals. The private nurses attended 874 cases last year, and the district nurses paid 44,710 visits in Derby, and 20,351 in the out-lying districts. The opening of the Nightingale Nursing Home has more than fulfilled the expectations of the Board of Management. Two of the nurses and 7 pupils passed the examination of the Central Midwives Board during the year, and two of the latter qualified as monthly nurses.

### SHORT ITEMS.

The address of the offices of the Central Midwives Board, on and after June 23, will be Caxton House, Westminster, S.W., instead of 6 Suffolk Street, Pall Mall.—A large number of poor women attended the funeral service of Miss Blanche Maddox (better known as Nurse Blanche) last week at Balham, in order to testify their respect to the memory of the deceased, who, having been through a course of training at York Road Maternity Hospital, worked for several years with great devotion in the squalid streets of the Borough.

### The Nursing Outlook.

"From magnanimity, all fears above;
From nobler recompense, above applause,
Which owes to man's short outlook all its charm."

### A GREAT NURSE-TRAINING SCHOOL.

### I. NUMBERS AND GROWTH.

WE spoke last week of the privilege we had enjoyed of reading the reports of the matron of the largest training school in the country. We referred of course to the London Hospital where Miss Lückes has discharged the duties of matron for upwards of 25 years to the entire satisfaction of the governors, and with marked resolution and gratifying success in many directions. Miss Lückes has always held her own independent views on nursing matters and she has never deviated from the principles which she held at the outset to be essential to the successful administration of a training school for nurses.

The school at the London Hospital was started in 1880, and certificates of two years' training have been granted to 1,473 nurses during the period of its existence. The Nightingale School was founded in 1860, and the number of nurses certificated by them as having completed one year's training is 1,097 . In this connection it is necessary to qualify the exaggerated statements often made as to the total number of nurses, that is of certified nurses. The latest instance being that of Miss Clark, who, at the meeting of the Royal British Nurses' Association reported last week, expressed herself as being a responsible representative of 80,000 nurses working all over the kingdom. The evidence given before the Royal Commission, and the figures and facts submitted to that Committee, showed that, under the most liberal estimate, there cannot be more than from 25,000 to 30,000 nurses in this country at the present time. We incline to think that these are outside figures, seeing that if the oldest training school, St. Thomas's and the largest training school, the London, have produced far less than 3,000 certified nurses, about half of whom, speaking roughly, had one year's training, and rather more than half two years training in 43 and 23 years respectively, it stands to reason that, taking all the other training schools in London and throughout the country, they cannot have produced anything approaching the same numbers as the two schools mentioned, when they have enforced three years' training before granting their certificates. Of the certified nurses who have passed through the schools, a very considerable number must have left the nursing ranks long ago, so that the actual number available for nurses' duties at one time, and especially at the present time, cannot equal anything like the actual numbers who have gained certificates. Many have died, married, or given up nursing for a variety of personal reasons. Many others have relinquished the work from a breakdown in health, not necessarily in consequence of their nursing work, though, of course, some instances of that are known, but because out of so large a number of women engaged in any occupation, a certain proportion must certainly become incapacitated by illness. Another factor affecting the actual number of working nurses available for service in this country to-day is that the number of nurses certificated by the schools has been relatively few until recent years, and that of this number many nurses remain longer in the service of the hospital where they were trained than the actual period required to complete their training. The numbers certificated is reduced, too, by the circumstance that large numbers of nurses who enter a training school leave for a variety of reasons without completing their training. We have been dealing with certified nurses only, because the whole trend of the present day is to improve the training by raising the standard of education, and so to limit the title of trained nurse to those only who have obtained the recognised certificate.

The first point which must strike anyone who studies the working of the London Hospital training school is the enormous growth in the volume of work which has devolved upon the matron and her staff, especially in later years. Thus in 1893, when the in-patients numbered 10,599, the total number of the nursing staff at the London Hospital was 275. In 1905 the in-patients amounted to 13,552, but the complete nursing staff has increased to 631 nurses. The private nursing staff in 1893 numbered 45, but it had increased in 1905 to 200 nurses, a fact which testifies eloquently to the volume of service rendered to the public at large by the authorities of the London Hospital. Deducting the private nursing staff, it will be seen that the hospital staff of nurses and probationers has nearly doubled in the 14 years, although the increase in the number of in-patients treated has been about 30 per cent. only. These figures illustrate the enormous development in treatment and the marked change which has taken place in the character of the cases admitted to hospital beds in London in recent years.

Apart from the growth of mere numbers, the actual severity of the cases admitted is much greater than formerly, and the increase in the number of operation cases is stupendous. The effect of the latter circumstance upon the nursing work of the London Hospital may be gathered from the fact that there are about 81 operations a week, and that about 50 per cent. of the nurses engaged in the wards during the day are required for operation cases alone. Anyone with a knowledge of the difficulties which a matron has to face in ordinary circumstances will appreciate the strain which the head of a great nurse-training school must experience in order to meet the nursing requirements of the surgical staff of a great hospital. It is not easy to convince a committee that they must grant the necessary funds in order to secure an adequate supply of nurses to meet the ever-increasing demands of the medical staff for their services year by year. That Miss Lückes has succeeded in supplying all the requirements of the London Hospital for upwards of 25 years with efficiency and completeness is a striking testimonial to her merits and great administrative gifts.

### The Care and Nursing of the Insane.

By Percy J. Baily, M.B., C.M.Edin., Medical Superintendent of Hanwell Asylum.

### I.—ANATOMY AND PHYSIOLOGY.

(*Continued from page* 133.)

*The Act of Swallowing.*—When the process of mastication is complete the food is rolled into a ball by the tongue and is passed to the back of the mouth by the muscular (voluntary) action of the tongue acting against the hard palate. In other words, the bolus of food is simply squeezed backwards between the surface of the tongue and the palate. It is prevented from passing back into the nose (through the posterior nares) by the muscles of the soft palate. When it reaches the back of the tongue the bolus is grasped by the voluntary muscles of the pharynx or throat and at the same time the larynx (the upper portion of the air passages) is drawn up towards the base of the tongue, which in its turn is depressed to meet it. In this way the entrance to the air-passages (glottis) is closed and the food is prevented from passing down them. In various paralytic diseases such as general paralysis or some forms of paralysis resulting from localised brain-softening, and also after diphtheria, the soft palate and the muscles concerned in closing the glottis may be paralysed. In these cases the food is very liable to pass back into the nose or to get down into the air-passages and cause choking.

By the contraction of the muscular wall of the pharynx the food is forced downwards into the gullet. Up to this point the act of swallowing is purely voluntary. When the food reaches the gullet, however, the muscles which force it onwards are all of the involuntary kind, and the remaining stage of the act of swallowing is entirely involuntary. The bolus of food, having reached the gullet cannot be recalled by an act of the will, but must pass onwards into the stomach.

When the food reaches the stomach it is freely mixed with the *Gastric Juice.* The gastric juice is the secretion of the glands which lie in the mucous membrane of the stomach. It is an acid secretion, and its function is to dissolve or digest the *Nitrogenous portions* of the food—that is to say, the fleshy or meaty parts of our diet—converting them into *Peptones.* This particular action of the gastric juice depends entirely upon the presence in it of a ferment which is called *Pepsin.* We have seen that the presence of food in the mouth stimulates the activity of the salivary glands, so that saliva is abundantly poured forth. It is the same with the stomach. When the organ is empty its mucous membrane is relatively pale, and the secretion from it only scanty. On the entrance of food, however, the amount of blood which passes to the stomach is enormously increased, and the secretion from the peptic glands becomes abundant. This increased activity of the stomach commences as soon as the food enters the mouth, and is a nervous phenomenon.

In addition to pepsin, the gastric juice also contains another ferment called *Rennet.* Rennet curdles milk, and hence, when vomiting occurs soon after the patient has taken milk, the milk is always curdled.

Under ordinary circumstances, when the food has been thoroughly masticated, peptic digestion occupies a period of from three to four hours. During the whole of this time the involuntary muscles which, as we have already seen, exist in the walls of the stomach are slowly contracting and relaxing, so that the contents of the stomach are kept continually moving, and are thus churned up and thoroughly mixed. The food in this state is known as *Chyme.* While gastric-digestion is going on the chyme is kept in the stomach, and is prevented from passing on into the intestine by the contraction of the muscular fibres of the pylorus. When the process has been completed in the stomach these muscular fibres relax and the chyme passes through the pylorus into the first part of the small intestine or duodenum.

In the duodenum the food is mixed with the secretions of the liver (bile) and pancreas (pancreatic juice), and undergoes a complete change in its character. From being a yellowish acid fluid resembling pea-soup as the chyme is, it becomes milky and alkaline, and is now called *Chyle.* These changes are brought about chiefly by the action of the bile and pancreatic juice.

The *Bile* is a yellowish or reddish green bitter fluid of slightly sticky consistence. Alone it has but very small influence upon digestion, but when mixed with pancreatic juice it greatly assists this in some of its functions. When the bile, however, is prevented from entering the intestine, as may be the case in certain states where jaundice occurs, the motions lose their natural colour and become whitish or resemble clay; they also have a very foul odour and much gas is generated in the gut. Hence bile is thought to exercise some antiseptic property in the bowel. The only food-stuffs which are really acted upon by the bile are fats. These it partially converts into soap.

*Pancreatic Juice* is a colourless fluid much resembling saliva. It is a very powerful agent in digestion and attacks—

1. The starches, converting them, like saliva, into sugar.
2. Nitrogenous foods or albumins, and like the gastric juice, it converts these into peptones.
3. Fats. These it emulsifies.

The fats of our food all melt at the temperature of the body and assume a condition like oil. By the action of the pancreatic juice this oil is divided into minute globules, which float in the chyle and gave it its milky appearance. Fat in such a finely divided state is said to be emulsified. Milk is the best-known example of a natural emulsion, and we know that the fat of the milk (cream) takes some hours to separate out as a distinct layer on the top. Fats emulsify much more readily in the presence of small quantities of soap, and hence the emulsification of the fats of the food is hastened by the fact that the bile in acting upon the fats partially converts them into soap in the duodenum.

As the food passes on through the small intestine it is further mixed with the secretions which pour out of the glands which lie in the mucous coat of the gut (the *Intestinal Juice*). This also has some digestive power and probably resembles the pancreatic juice in its action, although it is comparatively feeble. With the small intestine the process of digestion ceases, and when the intestinal contents enter the colon no further digestive change occurs in it.

As in the stomach, the food in the intestine is continually being moved and churned about and, at the same time, forced onward towards the rectum by the alternate slow contraction and relaxation of the involuntary muscular layer of the intestinal wall.

After this short survey of the changes the various food stuffs undergo in the alimentary canal in order to render them fit for absorption, and of the agencies or juices whereby these changes are effected, it only remains now to add a few words to explain how the food thus prepared is absorbed and ultimately reaches the blood.

The mineral portions of the food (common salt, bicarbonate of soda or baking soda, phosphates of lime and magnesia, etc.) are readily soluble in the fluids of the mouth and stomach, and are absorbed together with the water directly into the blood without undergoing further change. The *sugar* which results from the digestion of starches, and the *peptones* which result from the digestion of nitrogenous foods are also absorbed directly into the blood. The absorption of these substances commences in the stomach, and continues as the food is passed on through the intestines as far as the rectum. We have seen that digestion ceases when the food leaves the small intestine, the products of digestion, however, still continue to be absorbed from the contents of the large bowel.

These digested matters (note that we are not speaking of fats now) pass into the blood which circulates in the capillaries of the mucous membrane of the stomach and intestines. From these capillaries, as we have already seen, the blood is collected by the portal vein, and is by it carried to the liver. After passing through this organ it is thrown into the general blood current on its way to the heart in the inferior vena cava.

The emulsified fats are not absorbed directly into the blood by the blood-vessels. These pass into the lymphatic vessels or lacteals of the intestines. We have seen that the mucous membrane of the small intestine is studded with an enormous number of minute finger-like projections called villi. Each villus contains, in addition to blood capillaries, the commencement of a little lymphatic vessel. Into this vessel the emulsified fats are received. They then enter the general lymphatic stream, and are conveyed by the thoracic duct into one of the large veins at the root of the neck.

Those portions of the food which, after being acted upon by the various digestive juices, still remain insoluble, pass on through the large intestine into the rectum, where they are discharged as fæces through the orifice of the anus.

### The Nurses' Clinic.

### ABDOMINAL INJURIES.

Of all cases requiring careful surgical nursing there are none which need more than those patients suffering from injuries of the abdomen.

There is often so little external evidence of severe internal injury that unless the patient shows signs of shock and collapse to a marked degree one might be tempted to think that he is not seriously hurt. Whenever the history suggests a probable internal injury the nurse must use all her powers of observation, so as to be able to report correctly and concisely to the surgeon if required.

When the patient has been put to bed, with a hot blanket tucked round him and hot bottles to the feet, he must be kept very quiet, and not allowed to exert himself in any way.

The position of the patient in bed must be decided according to the injury. If he is suffering from rupture of the rectus muscle, he will be best raised, so as to relax the abdomen, and thus bring the injured muscle into the best position to allow of healing again. If the patient is suffering from internal hæmorrhage and collapse, keep him flat, and raise the foot of the bed by means of a block, which will be found in all accident wards for this purpose.

The injuries may be "penetrating," such as are produced by some sharp instrument or a fall upon spikes or nails; or they may be "non-penetrating," such as are the result of a blow or kick from a horse, or of being run over. In these latter cases there may be little to show where the exact spot of the injury is, but this will not lessen the danger of internal mischief, and an exploratory operation may be performed to ascertain the amount and kind of injury should the patient's condition allow, and, if possible, to check hæmorrhage by finding and ligaturing the bleeding point. The patient will be in a state of extreme collapse if the hæmorrhage has been profuse, and the necessary apparatus for infusion of saline should be at hand ready for use at any moment. Application of external heat, raising the foot of the bed, bandaging the lower limbs and abdomen, are all useful in assisting the promotion of a better condition.

In assisting at these abdominal operations there will be much for the nurse to do to prepare the needed lotions, and perhaps administer stimulants by means of hypodermic injections of strychnine, brandy, or ether; but, although much has to be done, there will sometimes be a chance of noticing how the operation proceeds and the various methods employed, according to the injury or the method of the surgeon. In cases where the intestine has been torn or bruised and a "Murphy's button" has been inserted, the nurse must observe—after ten or twelve days—whether the "button" is passed in the fæces, and report it to the doctor.

Where the kidneys have been injured there will be blood in the urine, a specimen of which must be saved each day for examination. These patients must be kept lying flat and very quiet, and the diet must consist of cool or cold fluids. Do not allow the patient to lift himself, for the first few days anyhow; and he must stay in bed, even after the hæmaturia may have disappeared, some days. An instance

**THE NURSES' CLINIC—*Continued.***

of the importance of this may be seen in the case of a child admitted to hospital with an injury to the right kidney from a fall. He was treated in the ordinary way—i.e. perfect rest and fluid diet—and was allowed to get up on a couch and have thin bread-and-butter, the hæmaturia having disappeared two or three days before. After getting up there was again hæmaturia, and, after staying in bed a few days longer, it again completely disappeared. This time a week was allowed to elapse before the child got up again, and there was no return. This shows plainly that a lengthy rest in bed is necessary, and this rule must be adhered to if the result is to be a final and complete cure.

While mentioning abdominal injuries something may be said on the subject of fracture of the pelvis, which is sometimes a complication of injuries of organs within the pelvis.

The chief treatment for this will be absolute rest in bed. A firm bandage—a roller towel is excellent—is usually put round the pelvis and the patient kept flat on his back, with a good deal of attention to the back to prevent soreness. These are heavy cases to nurse, because of the lifting necessary for two or three weeks, until the patient may lift himself a little.

Where there is rupture of the bladder an operation will be necessary to sew up the tear, and a catheter will probably be left in to prevent extravasation of urine through the wound. The dressings in these cases must be kept as dry as possible, which means frequent changing, in order that the process of healing.

The feeding of patients suffering from abdominal injuries will obviously be a matter of care. Fluids will be invariably ordered where anything at all may be taken by mouth. In cases where an operation has been performed the doctor will decide the quantity, or, if the stomach or small intestines are to be allowed to rest for a time, rectal feeding will be resorted to. The preparation of these feeds varies tremendously; as a rule the surgeon says which of the many preparations he prefers.

Unrestricted fluids are usually given to those patients suffering from loss of blood, and injections of saline both per rectum and axilla greatly assist in the re-establishment of the normal supply of blood, and the patient's general condition naturally improves thereby. Stimulants are to be avoided in cases of hæmorrhage, unless specially ordered when there is severe collapse or shock or impending death.

There are few hard-and-fast rules which one can lay down with regard to the treatment of these patients, because injuries and the methods of treating them vary so much; though the foregoing hints may be useful, and may be adopted in cases where discretion allows. But the chief points to remember are those of keen observation, carefulness, and discretion, and to keep the patient at rest and as quiet as possible.

### The Royal National Pension Fund for Nurses.

### REPORT OF THE NINETEENTH ANNUAL GENERAL MEETING.

The nineteenth annual general meeting of the members of the Royal National Pension Fund for Nurses was held at River Plate House, Finsbury Circus, London, on Wednesday, June 6th, 1906, under the presidency of Everard A. Hambro, Esq., the Chairman of the Council. In addition to the members of Council and Governors present there was a large attendance of policy-holders, including the matrons of several important London, provincial, and Scotch hospitals.

Mr. E. A. Hambro: Ladies, it is my duty to ask you to receive the report and balance-sheet for last year, but before I absolutely ask you to do so I should like to make a few remarks on the balance-sheet. When I spoke to you last I tried to impress upon you all that the Royal National Pension Fund for Nurses was in no sense a charity, that it was a work of your own, that it was a work which would prosper under your hands, and that it entirely, or very largely, depended on your efforts whether it should go forward on its career.

### Sympathy of their Majesties.

When I say that besides being your own work, you have been very largely assisted in some respects, I would first bring to your memory how much their Majesties have helped you with their sympathy, and more especially in allowing you to call yourselves the Royal National Pension Fund. The hearts of their Majesties are always going out to everyone of their subjects, but I think they more especially go out to the nurses of the country. (Hear, hear.) I do not wonder at this. They have both themselves suffered, and must both themselves have appreciated the enormous advantage that a well-trained, sympathetic, and kind nurse can bring to the bedside. I think that whenever the history of this reign is written some of its most prominent features will be in connection with the sympathy displayed by their Majesties to all in sickness or in trouble. I myself have appreciated the kindness of His Majesty. I was once ill in a foreign country, and he came and sat by me, talked kindly words to me, cheered on the nurse who was nursing me, and in every way comforted me. That which His Majesty did for me and did for you through raising the status of nurses, he has done, by assisting in promoting the welfare of the hospitals of the country, for every sick and suffering person in his realm. I would, therefore, ask you to record your vote of thanks to their Majesties for all the kindness they have shown to you and through you to all sick and suffering persons. May I not say that you all appreciate this? (Cheers.)

### Thanks to Secretary and Staff.

While I am on the subject of thanks, I should like to go a little further. There are other things for which you have to be thankful. I would ask you to convey your thanks in the same hearty way to the staff, and more especially to the secretary, for so ably carrying on your affairs.

### Appreciation of Mr. A. J. Waley's Assistance.

There is one other person who is always thinking and acting for you—I refer to the gentleman who so carefully goes through your securities at the end of each year, who devotes a great deal of time to seeing that they are as they ought to be, and who comes and talks to me whenever he sees anything which he thinks may be suitable for you. To-day, unfortunately, according to the Eton Calendar, is the 4th of June, and he has to attend, or else he would have been here. I would ask you to thank him. (Cheers.) I will now ask you to go to the report itself. I want you to notice the Pension Branch. The number of policies issued during the year was 1,304.

### Co-operation.

I will here say that I received a letter from your worthy secretary on this subject, which I will read to you. He says:—"There is little doubt that the large increase in the number of policy-holders last year was due in a very great measure to the increasing interest which the members are taking in the Fund; I think they are really beginning to understand what the meaning of co-operation is and that if they will try and get new members they will benefit themselves as well as those who come in." I want you to take this very much to heart. I want you to remember when you go home and are associating with other nurses to try and show them some of the benefits of the Fund.

### Benefits of Saving.

The rest of my remarks on the report will not be so much on £ s. d. as on what I consider some of the benefits derived from it. One thouand three hundred and four new policies have been taken out in the past year—savings. Saving seems very easy, but it is very difficult to perform; and I cannot help thinking that when you have a fixed date on which you have to save, it enormously assists in saving. If you have a certain amount of which you intend to save a portion, and you mean to put it, say, in the Post Office Savings Bank the next time you pass by it, it is not so likely to get there as when you have a date on which you have to make your saving. You save better when you have a real object—a real date on which you have got to save. I want you, therefore, to regard that as one of the benefits of the Fund, and as a help towards your saving. Then there were 419 withdrawals last year. This may sound disadvantageous to you, although it should have a good influence again as showing you that the Pension Fund does good. Four hundred and nineteen people who, for some reason or other, have desired to withdraw—most of them, let us hope, for the reason of matrimony, which is very often the reason given—have been able by their own exertions, whatever their reason was for withdrawing, to have a nest egg to take away. I am sure that your Pension Fund does good in that sense—that those who wish to withdraw are able to withdraw when they want, and for whatever purpose they want.

### Pensions Paid—£14,000 a Year.

Next I would call your attention to the pensions falling due. When I was young—alas! many years ago—nurses were sometimes forgotten by those whom they had nursed, or, if not forgotten, they had very little hope of an assured income for their services as time went by. You have now, thanks to your Pension Fund, 627 women drawing £14,000 a year. Is not that a satisfactory sum when you think that it is all owing to your connection with the National Pension Fund? And does it not show the good which the Pension Fund is doing? I venture to draw these three points to your minds because I always want you to remember that it is your Fund, and that its success depends on you—not on those who are on this side of the table. Now, I will go, perhaps, a little more into the £ s. d. question. The invested funds now amount to over £994,000—to be correct, £994,571 14s. 7d., on the date of this report. That is a large sum. It reads glibly, but you would find it very difficult to count if you counted it sovereign by sovereign. This Fund we have done our best to invest as well as we were able to. We have scattered your investments over a large number of securities. It is human to err, and sometimes an investment bought on one day does not seem so satisfactory on another day. But they have nearly all come round with patience, and all of them, with the exception, perhaps, of the highest class, have come to their pristine price and more. We were bound to take a certain amount of Consols for you, because every institution must hold them. That is the only security, I think, that shows any loss, but the others show so much more profit that it more than counterbalances the loss shown on what is commonly called the very best securities.

### New Offices.

There is one other security we have gone into, and it shows that your work has got so large that your offices are not capable of carrying it on properly. One hundred thousand letters a years are coming in and out of the office every year; many nurses come to ask questions; and the staff is hampered for room. We have, therefore, thought it wise (I do not myself understand about an investment in London property, but it is being done under the best advice)—we have thought it wise to buy 15 and 16 Buckingham Street, which is very convenient for nurses, having the Strand on one side of it and the Underground Railway on the other, with the station at Charing Cross. Here we hope the offices will be more commodious and also more handy to nurses (they will not be more handy to me), and we hope, by letting off that space which we do not want for you, we shall be able not only to get a good office, but to bring in interest on the money invested in it. Before I sit down I think I will remind you of a little story that happened out in the West. There was in a diggings town a saloon in which there was a little stage like this, with a piano on it, on which a man played. Above his head were these words: "Do not shoot the pianist. He has done his best." Well, I have done my best. I hope you will not shoot me, but that you will pass this resolution. (Cheers and laughter.) I move "That the report and accounts and balance-sheet be received and adopted."

Sir Henry Burdett, K.C.B.: I have much pleasure in seconding the adoption of the report. Mr. Hambro's excellent and interesting speech has covered most of the points, and there are only two points I wish to bring out.

### Sick Pay.

The first is as to the Sick Fund. It is a great disappointment to me that the nurses as a body—and especially those who are joining now—do not seem to appreciate the power and utility of the Sick Pay Branch. That branch was instituted in connection with this Fund in order that every worker who entered the field of nursing who chose could make herself perfectly safe in her working life and afterwards by joining the Pension Fund. I know that many of the nurses, if they are in institutions, think that they are provided for, and that sick pay is not a matter of importance; but it is, because underlying it there is a very important principle—that is, the principle of the independence of women workers, and sick pay is the bedrock on which every working nurse can stand secure if she will enter for it when she joins this Fund. Those of you who join may have all through your life the feeling that you are helping yourselves and that you are safe. A nurse may say that if she enters for sick pay for only 10s. a week it is a very small and inadequate sum; but in practice our experience shows that a woman who has to prematurely retire from work who has 10s. a week regularly coming in may be a person of consideration with her friends, and should find little difficulty in providing suitable accommodation for herself, when without it she would be probably in a very serious state. Then there is another feature of the Fund in this connection—the Benevolent Fund.

### Benevolent Fund.

It is when you fall out on life's journey, from no fault of your own, and are unable to continue your work, when your resources may not be adequate for your maintenance and to secure you the necessary treatment which you require, the Committee of the Benevolent Fund steps in, and every member of this Fund has the consolation of knowing that her circumstances will be fully gone into, and that she will not only get, through the Benevolent Fund, the necessary additional monetary support, but also the right hand of fellowship and all the sympathy, comfort, and aid which she most needs in her evil days of sickness or disaster. Therefore I venture to think that it is wise for nurses to join for sick pay, and that, in addition to what Mr. Hambro has so well said in favour of the Pension Fund, we have to thank Lady Rothschild and the ladies who so admirably manage the Benevolent Branch for supplementing the business side of the Fund, and making it the power for good which it undoubtedly is. I have much pleasure in seconding the adoption of the report. (Cheers.)

The Chairman put the motion to the meeting in the usual way, and it was carried.

### Policy-holders' Representatives.

He afterwards announced the result of the election of representatives of policy-holders as members of Council, and thanked the scrutineers, Mr. H. C. Pulley and Dr. Potter, for their services. The voting gave to Miss Mabel Cave 1,042 votes, Miss P. Peter 1,036 votes, Miss Chambers 706 votes, and to Miss Macfarlane 690 votes; and declared Miss Cave, Miss Peter, and Miss Chambers duly elected.

### Re-election of Members of Council.

Mr. George King, F.I.A., F.F.A., in moving the re-election of five members of the Council, said :—

### Origin of the Fund.

My memory is carried over very nearly twenty years, because it is twenty years ago this coming autumn that Sir Henry Burdett consulted me, and I first began to prepare the tables and settle this scheme, which has been in operation ever since. It was Sir Henry Burdett who conceived the idea of the scheme—(cheers)—and his enthusiasm has carried it through to its great success. That his name should be on the list twenty years after he called me in to advise in arranging the details of the scheme is a great pleasure to us all. The other gentlemen whose names are on the list are men of eminence, and their names among the members of your Council inspire confidence in the Fund. The report itself shows how great that confidence must be, and the Chairman's remarks confirm it. That we should have such a large number of nurses contributing their savings to this Fund for what, to many of them, would appear distant old age, shows what great confidence there is in it. That they should come in in increasing numbers, and that so few should leave again, shows that their confidence is not diminishing; and that confidence, I venture to submit, is inspired by the Council which guides the affairs of the Fund. (Hear, hear.) It is therefore a great pleasure to me to propose that these gentlemen be re-elected.

### Economical Management.

The management is shown to be first-class, even if you only look at the expenses of management. A Fund of this kind has more detailed work than almost any insurance company except, perhaps, the industrial companies; but I should like anyone to name an insurance company which manages its business on less than 4½ per cent. of its premium receipts. Yet that is what the Fund does, and it shows how carefully the Council must manage the finances that they should work so very cheaply. There are many other points that might be mentioned to show how excellent the management is, but it is really unnecessary.

Dr. George W. Potter : I have very great pleasure in seconding the motion, and, as you have had so many speeches, all excellent, I will not add another.

The motion was carried unanimously.

### Re-election of Auditors.

The Chairman put the motion for the re-election of Messrs. Whinney, Smith, and Whinney, at a remuneration of fifty guineas, and having declared it carried, inquired whether anyone present would like to make any remarks.

### Thanks to the Chairman.

Sir Henry Burdett : The resolution I have to propose is one which is not at all formal, and I hope you will consider it the resolution of the day. It is a vote of thanks to Mr. Everard Hambro for presiding on this occasion, and for his work as Chairman of the Council of your Fund. (Cheers.) This Fund owes much to Mr. Hambro. When I look back on the early days of Mr. King and I, in considering how we could find a unit or basis to build this Fund upon—I am conscious that Mr. Hambro had a much greater influence in establishing the Fund and getting it started than he is aware of. He was an intimate personal friend of Mr. Junius S. Morgan, who had a very sincere affection for him and a very high opinion of his judgment. It was to the encouragement I received from Mr. Morgan really that we owed the starting of this Fund—(cheers)—because unless we could have come under the Insurance Acts by depositing £20,000 we never could have incorporated it as an insurance company, and it would not have been in existence to-day. Mr. Morgan offered to give £5,000 if three others would give a like sum, and Mr. Hambro was the next city merchant who came forward and gave £5,000. (Cheers.) I am quite clear that that fact, coupled, as I say, with his friendship for Mr. Junius S. Morgan, had an immense influence upon the whole proceeding, and was, in fact, the underlying cause of much of the success which has attended the effort. When we get a little further and look down the years that have intervened, those most intimately connected with the working of the Fund know that Mr. Hambro has given of himself in working for you nurses in a way which has not only been immeasurably advantageous to yourselves, but has also been an example and encouragement to every one of us on the Council. We have the greatest esteem for Mr. Hambro, and the greatest appreciation of his work; and we can never forget that when we lost our first Chairman, Mr. Burns, he came forward and accepted the vacant office, although there was every reason for his refusing it, having regard to the claims upon him and the many other calls upon his time. Why did he accept it? Because he had the Fund at heart from the very first. He felt the Fund needed him, and he could not say no. (Cheers.) He said yes, against his own interest, and simply in testimony of the interest he took in the Fund. This proves how much Mr. Hambro had the Fund's welfare at heart. And with regard to your investments, Mr. Hambro has given his time week by week and year by year. It is to his great interest and personal care that the success of these investments is so largely due. I am sure, therefore, that this vote of thanks to Mr. Hambro to-day will be carried with acclamation, and with the utmost sincerity. I am also sure I may say on your behalf that we all wish Mr. Hambro long life and happiness when we tender him to-day our acknowledgments for the great services he has rendered to the Royal National Pension Fund from the day of its inception. (Cheers.)

### Funds—One Million Sterling.

Mr. T. C. Dewey (General Manager of the Prudential Assurance Company) : I should like, as one who very highly appreciates the work which has been done by Mr. Hambro for this Fund, to second the resolution. We are proud to have Mr. Hambro here to-day as Chairman, and I am quite sure that Mr. Hambro is equally proud to be present this afternoon, and to submit what is practically the record report of this institution. I say that it is the record report for three reasons—first, that our income from premiums has increased over the income of last year by 15 per cent., and for the first time exceeds £100,000; secondly, the pensions paid during the year have also increased by 15 per cent. over those paid in the previous year; and, thirdly, the funds have increased 10 per cent. over the amount of the previous year, and, for the first time, now exceed £1,000,000. And, notwithstanding the large increase that has been made—the record increase—the management expenses in amount are practically the same figure as in the previous year. I therefore say that it is our record report, and we are proud that Mr. Hambro is here to submit it. I beg to second the resolution.

Sir H. Burdett: I will ask you to signify your approval by your applause. (Cheers.)

The Chairman: Ladies, I am extremely obliged for the kind way in which the resolution has been proposed and seconded, and to you for the way you have received it. I can only say, as I have said before, that my best services are entirely at your disposal whenever you want them. (Cheers.)

The proceedings then terminated.

### Are Nurses Underpaid?

At the meeting of the Pension Fund, reported in another column, the following discussion took place:—

### Great Results Achieved.

Mr. Pollitt (Blackburn): Mr. Chairman, if I might take up a minute or two, I should like to do so. I am quite sure that this report must be most gratifying to all of the Council, to the Secretary, and to all the policy-holders. After nineteen years of work—which is really, as these matters go, but a short time—I cannot help feeling, as a very humble member of the Nursing Committee in Lancashire, that you have achieved very great results indeed; but, speaking with some recollection of what I know of our nurses in Lancashire, I cannot help saying that I think your results ought to have been very much greater—not that you could help that. The fact is that enough money has not come into your coffers to provide for the Nurses' pensions to the extent which should be the case. That is because nurses are not sufficiently well paid to enable them to support this Fund as it should be supported. In Lancashire we have some most curious views as to what £28 up to £40 a year will do. I know a very well-to-do lady who is perfectly aghast that £40 a year will not do everything for a nurse, although a nurse does not get £40 a year at first. But say £40, and it means 15s. 4d. a week. This lady cannot understand how it is that a nurse having £40 a year has not the money for almost anything, and a large sum for making provision for old age. I ventured to ask her if she had any idea as to what a nurse had to provide on £40 a year, and she did not seem to have much of an idea. She said that a nurse had her uniform provided and her food and house rent. I pointed out that a nurse had to do three things—to provide money for a month's needful holiday, to provide clothing other than uniform, and to provide all those little expenses through the eleven months of the year which a nurse must incur if she is to keep an edge on her health and to take a reasonable amount of pleasure. We see nurses going out frequently on bicycles, which may involve railway journeys. That may not amount to much, but it is something, and with an occasional cup of tea and other little things these expenses amount to a considerable sum after a time. This lady, it appeared, was told that it was not necessary for any nurse to spend more than £5 a month for her holiday if she would go on the Continent. With such ideas as these, she had got it into her head that £40 a year was a very handsome income. But I venture to say that it is not enough. I venture to say that something should be done now in the way of considering whether the time has not arrived for stirring up every Committee in the country to consider what can be done to enable nurses to make a really suitable provision. You have an average of £21 a year for 627 nurses. That is not very much. We heard from the gentleman who seconded the motion that 10s. a week was a very fine thing, and I do not wish to oppose anything he said; but, after all, 1s. 6d. a day is not very much to exist upon. The question is what should be done, and who should do it? I think myself that it is perfectly useless for the Committee of the Queen Victoria Jubilee Institute to write to the Committees in the country, and I also think that the right thing to do is to take steps which would interest the very highest in the land. If you have anything very important to do, it is always well to go to the chief men, and I would suggest in this case that the right thing is to interest the King, the Queen, and the Prince of Wales, and let us have some leaders in the *Times* and other daily newspapers, and let the public be educated to the idea that they are not paying for what they are getting in paying nurses the remuneration they are giving to their scullery or parlour maids.

### The Pay of Nurses.

I say that nurses are not paid properly—(cheers)—and that the time, in my opinion, has come—and I speak after my Lancashire experience—for increasing their pay, and if you increase their pay, your Fund, instead of being £1,000,000 would be £3,000,000, and instead of your annuity being £21 it would be £63, and £63 is little enough after the life that a nurse has lived. (Cheers.) That is what I think should be done, but who should do it? I think that the Council of this Fund contains the right gentlemen for doing what is needful. You can very properly say that you are not working for shareholders' dividends. You can very properly say, after nineteen years, that you have been working hard and have done well with what you had, but that you want more funds and more investments to manage. I think you should, as we say in Blackburn, "kick up a row," and bring those who are concerned in this matter to the view that they must do something. I hope that the result of whatever steps you take may be that the salaries of nurses all over the country will be doubled, at any rate, in a very short time.

Mr. E. A. Hambro: I am sure that we are all very much obliged to this gentleman for his kind remarks, and we all wish him success in his views. (Hear, hear.) I quite agree with him that the nurses in England are not paid in the way they ought to be paid, and that £21 a year, which is the average of the pensions I have enumerated, is not an adequate pension; but very great difficulties lie in the way of nurses improving their position. I do not advise them to strike, because I might be ill myself—(laughter)—but I really do not know any other way in which they could do it. Every sympathy I have would go out to do everything I could to assist in nurses' pay being put on a better basis, and if I can do anything to help them in any way my services will be always at their disposal in the future, as they have been in the past. Would no lady like to say anything?

### The Matrons' View.

Miss Morris (matron, General Hospital, Bristol): I should like to say a few words with reference to the remarks of Mr. Pollitt. I only speak as a matron of a provincial hospital. I am perfectly sure that if the general public would support the hospitals better the hospitals would pay their nurses better. But hospitals are not well supported, and, as we know, they are always in debt. Private nurses are well paid sometimes, but hospital nurses are not, and it is impossible for them to be well paid unless the public support the institutions better than they do. I should like to thank the Council for the great help they give us. I think it comes better from the nurses themselves to thank these gentlemen for the great interest they take in us and for getting our money out at the best advantage. I thank them very much myself, and I think I may do so on behalf of the nurses generally.

Miss Swift (matron, Guy's Hospital) : How to provide for those who are worthy yet growing too old to work, and who have made no provision for their old age, is a question which is engaging the attention of our Royal family, our statesmen, the managers of various public institutions, and not least, the generous managers of many of our hospitals. From me, as a member of the Junius M. Morgan Benevolent Fund Committee, and matron of a Metropolitan hospital, I hope that these few remarks on the subject of saving may not come amiss. On looking at statistics—the number of nurses passing into and out of our training schools each year in addition to the regular staff, and the number joining or belonging to the Pension Fund—the differences are appalling. I am sure we all agree that the secret of self-respecting independence in declining years is to commence early in life to make provision.

### Join the Fund.

Some who join our ranks have no need to make provision for the future, but I say to them, join the Pension Fund for example's sake. It is to matrons whom I would specially appeal, and as it cannot be a fact about us, as some venture to assert, that while women are clamouring for power, they are failing to see the many opportunities which lie nearest them, I claim that it is our special privilege as heads of training schools, large or small, not only to train women in sick nursing and ward management, but also to give them a fair start in life by our counsel and advice in matters of thrift and saving, for what can be sadder to realise than that our most worthy helpers are growing old, and that we have not done our part to save them from the humiliation of becoming dependent upon friends or the generous public? We know that our nurses as a class are hard-working, generous to a fault, self-sacrificing, but that they are not always practical. I claim that it is our duty to point out this moral and look ahead. I trust that we may be more successful in this respect in future, for we cannot fail to remember that we are training our successors, and so are making the future of our nursing world.

### Start Well by Saving Something.

Mr. E. A. Hambro : I beg to thank, and I am sure that we all thank, Miss Swift for her remarks. What she has said rather supports what I have always tried to impress on you—that the success of this Fund depends on you. If when you have the young under your care for training you will impress on them the necessity of laying by something, however small, although the total at the end may not be very large—as has been said—yet I am sure that the seed inculcated in their youth will induce them to go on and enable some future Chairman to announce that the Royal National Pension is not an average £21 a year, but very much larger.

Sir H. Burdett : Before I move the resolution that has fallen to my share I wish to answer the gentleman from Lancashire, and to point out that all I said was that a woman with 10s. a week in cash coming in regularly was in an infinitely stronger and better position than a woman who has not got it, and that 10s. a week is the minimum that a nurse should try and secure, whether she is in a hospital or in private practice. As to the average of £21 a year as a pension, you must remember that that average is worked out on all our policies, and experience shows that if once a nurse begins by entering for a small policy, she very soon takes out another policy if she prospers, and in that way there are some nurses in this Fund who may have a pension when they retire of from £50 to £100 a year. That is the practice, and that is how the early savings may work out.

### Laying the Foundation-Stone of the Nurses' Home at University College Hospital.

The block of buildings which include the School of Advanced Medical Studies, Nurses' Home, and Maternity Students' House, of University College Hospital, has already risen a good height from the ground, and those who attended to witness the laying of the foundation-stone for this block by Sir Donald Currie on Monday last found themselves ushered into the future library. This spacious room boasts of four walls and a ceiling, but all very much in the rough as yet. The foundation-stone, bearing a fitting inscription, was let into one side. The room was decorated with flowers and bunting, and when the guests, in bright summer dresses, and the doctors in their richly hued hoods, filled up the seats it looked anything but bare. Up in a gallery the band of the Scots Guards were ensconced, and a block of seats at one end was filled with nurses to the number of about 50. Two pretty little maidens, Miss Olive Martin and Miss Marjorie Johnson, presented Lady Currie with a bouquet of dark crimson carnations and lilies of the valley, and Sir Donald with a magnificent pink carnation for his buttonhole.

Sir E. Busk, Vice-Chancellor of the University of London, addressed Sir Donald Currie in the regretted absence of Lord Rosebery, and briefly sketched the state of affairs when Sir Donald Currie came to the rescue of the authorities with his munificent gift of £100,000, of which £20,000 was to provide a nurses' home in connection with the hospital. To this sum Sir Donald Currie's three daughters contributed £2,500 for the furniture of the Home.

Lord Fitzmaurice, on behalf of University College, and the Duke of Bedford as President of the Hospital, thanked Sir Donald Currie warmly for his generosity.

Sir Donald Currie, who was greeted with loud and prolonged applause, thanked them for their very kindly reception of him, and said how gratifying it was that the buildings would be erected free of debt, and that there would be a balance of £20,000 in hand for the upkeep of the medical school and the Home. It was greatly to be regretted that there was so much difficulty in obtaining support for the many hospitals of this country, and that these institutions might soon have to be supported out of the rates. After touching upon the medical school and the problem of its subsistence, he went on to say that he rejoiced to know that the Nurses' Home, providing as it would accommodation for about 70 nurses, was destined to fulfil all the expectations entertained with regard to it. He knew the value of good nursing, and they must all acknowledge how important it was that the nurses should have a comfortable home, and in close proximity to the hospital. It had been a great pleasure to himself, and to those connected with him, to be able to add sufficient to fit out the Nurses' Home in addition to the building.

The ceremony of laying the foundation-stone was then performed, and afterwards the whole company adjourned to the grounds of the College, where tea was served.

### Wants and Workers.

Will anyone, having a tent or awning, give it for the use of a poor consumptive, so that he might be out of doors? Carriage would be paid by Nurse Farrell, Studley R.S.O., Warwickshire.

### Appointments.

Cosmopolitan Hospital, Venice.—Miss E. A. Chaffey has been appointed matron. She was trained at the South Devon and East Cornwall Hospital, Plymouth, where she was afterwards sister. She has been sister at the Hospital for Sick Children, London; served in the field during the South African campaign; and has done private and district nursing.

Derbyshire Royal Infirmary, Derby.—Miss Gibson has been appointed night superintendent. She was trained at the Great Northern Central Hospital, Holloway, London, and has since been sister at the Grimsby and District Hospital. She has also done private nursing.

East Suffolk Hospital, Ipswich.—Miss Annie Butcher has been appointed sister. She was trained at the Royal South Hants Hospital, Winchester, and has since been charge nurse at Baskitt Hospital, Barnsley; nurse at the Jamsatjee Jeejeebhoy Hospital, Bombay, and the Jessop Hospital for Women, Sheffield. She holds the certificate of the Central Midwives Board.

Eliot Cottage Hospital, Haywards Heath.—Miss Mary Barrett has been appointed nurse matron. She was trained at the Middlesex Hospital. She has since been sister of the women's wards and sister of the men's wards, with charge of theatre and casualties, at the General Hospital, Tunbridge Wells; and ward sister at Kingston Poor-law Infirmary. She has also done private nursing in Brighton. She holds the certificate of the Central Midwives Board.

Hammersmith Infirmary, Ducane Road, W.—Miss Lilian Piper has been appointed assistant matron. She was trained at Leeds General Infirmary, where she was afterwards nurse and temporary sister. She has since been charge nurse at a Small-pox Hospital, the Park Fever Hospital, and the Brook Hospital, under the Metropolitan Asylums Board; she has done private nursing in connection with Miss Mocatta's Home at Southampton; and she has been night superintendent at Islington Poor-law Infirmary.

Infirmary, King's School, Canterbury.—Miss B. X. Hall has been appointed nurse in charge. She was trained at the Royal Infirmary, Sheffield. She has since been sister of a male accident ward at the Samaritan Free Hospital, London; charge nurse under the Metropolitan Asylums Board; nurse-in-charge of the Infirmary of the Royal Masonic School for Boys, Wood Green, London; and nurse-in-charge of the Infirmary of the Royal Naval School, Eltham, Kent.

New Hospital for Women, Euston Road.—Miss Elizabeth Williams has been appointed charge nurse. She was trained at the United Hospital, Bath. She has since been staff nurse at Llandrindod Hospital and sister at St. Mary's Infirmary, Islington.

Portsmouth and South Hants Eye and Ear Infirmary.—Miss Dora Clay has been appointed matron. She was trained at the Cumberland Infirmary, Carlisle, and has since been charge nurse at Park Hospital, Lewisham, and ward and theatre sister at the Royal Portsmouth Hospital.

Royal Infirmary, Derby.—Miss F. M. Gibson has been appointed night sister. She was trained at the Great Northern Central Hospital, London, and has since been sister at Grimsby and District Hospital.

Royal United Hospital, Bath.—Miss Fanny Louisa Mason has been appointed sister of the men's surgical wards. She was trained at the Royal United Hospital, Bath, where she has since been staff nurse. She was subsequently night superintendent at St. Mark's Hospital, City Road, London.

### Everybody's Opinion.

[Correspondence on all subjects is invited, but we cannot in any way be responsible for the opinions expressed by our correspondents. No communication can be entertained if the name and address of the correspondent are not given as a guarantee of good faith, but not necessarily for publication. All correspondents should write on one side of the paper only.]

### NURSING AND NURSING HOMES.

Nurse Harty, of Aigburth, Liverpool, writes: Referring to the "Note" in your last issue, I think it should also be made known that my defence at court was that I had considered myself justified in breaking my agreement because first the plaintiff had completely broken each agreement with me. Also that her counsel himself proposed that they would pay all costs of the case if I were willing to give up nursing in St. Annes, to which I agreed. I trust that my experience may bring up the question: Why should the custom be for a matron to have a legal agreement and the nurse only a verbal one? for it must be admitted that just as there are nurses with elastic consciences—and, alas! some with none at all—so there are matrons, and both should have protection from each other.

### HELP FOR SICK CHILDREN.

Miss G. Gordon Smith writes from Boyne Towers, Tunbridge Wells: Having been a constant reader of your most interesting and valuable paper The Hospital for about ten years, I am wondering if you could help me through its columns. I am in charge of the district of Walworth and part of the Borough for the Invalid Children's Aid Association, and am terribly in want of visitors. There are about 100 sick children in my district, some of whom are very ill, and unable from one reason or another to attend the special schools of the London County Council. These urgently require individual visiting, and in some cases teaching; though I have a trained nurse working for me we cannot possibly give the children the individual care they so much require and would so appreciate. To a trained worker especially the work is almost of boundless scope, and indeed requires developing in many directions; but it seems almost impossible to hear of any ladies willing to visit these children regularly and systematically. If necessary, I am willing to pay the expenses of any visitor, and would give full particulars to any lady who would be kind enough to help.

### TRAVEL NOTES AND QUERIES.

By our Travel Correspondent.

Austrian Tyrol and Italian Dolomites Tour.—Nurses whose holidays fall at the end of June will be glad to learn that a tour for gentlewomen has again been organised by Miss L. M. Davidson, whose name is familiar to the readers of The Hospital, to start on June 27th, for 35 days, to be spent in the Austrian Tyrol and the Italian Dolomites. Amongst the places to be visited are Bale, Zurich, Innsbrück, Tolbach, Cortina, the Ghedina Lakes, Peravolo, etc. The terms (31½ guineas) are strictly inclusive, and allow for first-class on the steamer, reserved second-class in the train throughout, and three meals a day in addition to afternoon tea. All particulars can be had from Miss L. M. Davidson, 25 Marjorie Grove, Lavender Hill, S.W.

On the South Coast (Ethel).—Hydros are not usually met with on the coast. I think that Folkestone would be the sort of place you want; it is always lively, and yet you can be quiet if you wish. There are many boarding-houses. Write for terms to "Richmond House, Clifton Crescent," "St. Osyth," "Westcliff Boarding House," and "Kentish Hotel, Alexandra Gardens." This last is the cheapest. Then how would Bognor do? It is a little further off and not so lively. Write for terms to Mrs. Coutts, The Campo, Aldwick Road West, and Mrs. Tufnall, The Arlington, West Parade. I know of no nurses forming such a party as you wish.

### Notes and Queries.

**REGULATIONS.**

**The Editor is always willing to answer in this column, without any fee, all reasonable questions, as soon as possible.**

**But the following rules must be carefully observed.**

1. **Every communication must be accompanied by the name and address of the writer.**
2. **The question must always bear upon nursing, directly or indirectly.**

**If an answer is required by letter a fee of half-a-crown must be enclosed with the note containing the inquiry.**

*Cost of Uniform.*

(140) What does the uniform of a nurse usually cost?—*Ignorant.*

If you are entering an institution where uniform is not provided you had better write to the matron. She usually states what is required.

*Roman Catholic.*

(141) I am a Catholic. Can I enter a Protestant hospital without any disagreeableness?—*Inquirer.*

Most large hospitals do not object to receiving Roman Catholics, and you would meet with no disagreeableness.

*Nursing in London.*

(142) I am a trained nurse, and wish to join a nursing institution in London. Is there not a co-operation for nurses? *Lavender.*

Yes. The Nurses' Co-operation, 8 New Cavendish Street, W.

*Midwives Act.*

(143) Where can I see the Midwives Act?—*Mater.*

Write to The Scientific Press, 28 Southampton Street, Strand, W.C.

*Where to Train.*

(144) Will you advise me where to train.—*Nicholson.*

The last-named hospital in your letter is a splendid training school, and you had much better send in your application now, and then you may be accepted when you are 23. There are always a large number of candidates. See also "How to Become a Nurse," published by the Scientific Press, 28 and 29 Southampton Street, Strand, London, W.C.

*Village Nurse.*

(145) I accepted an appointment as village nurse, with three months' notice on either side. I was taken ill, and have now received one month's salary in lieu of notice. Is this legal?—*C. M. B.*

This question can be best answered by a lawyer.

*Medical Electrician.*

(146) Can I be instructed in the use of the catheter?—*Masseur.*

You can only secure this instruction through the kindness of some professional friend, or probably a medical man under whom you work would help you.

*Home Wanted.*

(147) Where can an old lady, age 82, able to walk a little, be received?—*Hampstead.*

Write to the Sister-in-Charge, St. Peter's Home, Kilburn, N.W.

*Nursing Abroad.*

(148) Can you tell me of any institutions which send private nurses to Nice for the season?—*Maude.*

No institutions send nurses to the Continent, but there is a Nursing Institution with English nurses at Nice.

*A Consumption Hospital.*

(149) Is the Royal Sea Bathing Hospital a consumption hospital?—*A. H.*

It receives scrofulous patients and those who have tuberculous joints, etc.

*Improver.*

(150) Where can I secure an appliance which will conceal the result of a cancer operation?—*Reader.*

Any corset establishment will supply you if you describe your want.

**Handbooks for Nurses.**

| | Post Free. |
|---|---|
| "How to Become a Nurse: How and Where to Train." | 2s. 4d. |
| "Nursing: its Theory and Practice." (Lewis.) ... | 3s. 6d. |
| "Nurses' Pronouncing Dictionary of Medical Terms." | 2s. 6d. |
| "Complete Handbook of Midwifery." (Watson.) ... | 6s. 4d. |
| "Preparation for Operation in Private Houses." ... | 0s. 6d. |

**Of all booksellers or of The Scientific Press, Limited, 28 & 29 Southampton Street, Strand, London, W.C.**

### For Reading to the Sick.

SUPPLICATION.

Jesu, Prince of life and light,
Dwelling now in glory bright,
Ruling all things by Thy might,
Hear us, Holy Jesu.

Thou Whose Death did death destroy,
Who through pain didst pass to joy
Endless and without alloy,
Hear us, Holy Jesu.

Thou Who didst to Heav'n ascend
Still to be the sinner's Friend,
Still Thy people to defend,
Hear us, Holy Jesu.

*T. B. Pollock.*

"That I may know Him, and the Power of His Resurrection."—Philippians iii. 10.

St. Paul is looking back, as he did more than once, on the great loss of his life—that he had not known Christ earlier. He had been no partaker in the wondrous scenes of the first Easter Day; nay, he had set himself to prove its impossibility. He remembers how he had hunted down those who had received the glorious tradition of the day of triumph. And it was only as a late comer, and in a strange and painful way, that he had met Jesus Whom he persecuted, and had seen Him in the agonies of a sudden conversion, and realised at length the depth of his error, and the intensity of his loss.

"That I may know Him."

What did he mean? St. Paul is exhibiting that which is such a wonderful characteristic of Christianity, the love for Jesus Christ as a personal friend, which has always been the mark, sometimes more, sometimes less, of the true Christian.

The Resurrection of Christ is no mere marvel, struggling to exact credence, in the dim past of history. It is the extension of the personal life of Jesus Christ here on earth to be the Living Friend and Companion of all, who in all ages seek Him and claim His love. To St. Paul He was just as close and just as real as He was to the disciples who were gathered round Him at the Last Supper, as dear as He was to the beloved disciple who leaned on His breast; as dear as He was to St. Peter, who tasted His forgiving mercy; as close as He was to the disciples as He walked with them on the road to Emmaus; as real as He was to that little band gathered in the Upper Chamber for the devotions of the first Christian Sunday.

What earnestness, what hope there is in this yearning longing, "That I may know Him and the power of His resurrection"!

Even while he writes these words he is a prisoner, and what imprisonment must have meant to a vigorous mind like St. Paul's we can only faintly imagine; and yet it is in this letter that he pours forth the most ardent signs, not of resignation, but of joy—"Rejoice in the Lord alway, and again I say rejoice." It is in this letter that he speaks of "the Peace of God which passeth all understanding," as keeping the heart and mind.

To him the Resurrection had widened the whole outlook of life. He had grasped the conception of immortality; he had bridged the grave; he had mastered disappointment; and opened the prison door, in the wide outlook of an endless life, which came to him from the Resurrection of Jesus Christ.—*Canon Newbolt.*

